# Characterization of Growth and Metabolism of the Haloalkaliphile *Natronomonas pharaonis*

**DOI:** 10.1371/journal.pcbi.1000799

**Published:** 2010-06-03

**Authors:** Orland Gonzalez, Tanja Oberwinkler, Locedie Mansueto, Friedhelm Pfeiffer, Eduardo Mendoza, Ralf Zimmer, Dieter Oesterhelt

**Affiliations:** 1 Department of Membrane Biochemistry, Max-Planck Institute of Biochemistry, Martinsried, Germany; 2 Institute for Informatics, Ludwig-Maximilians-University Munich, Munich, Germany; 3 Institute of Mathematics, University of the Philippines, Diliman, Philippines; 4 Physics Department and Center for Nanoscience, Ludwig-Maximilians-University Munich, Munich, Germany; University of Illinois at Urbana-Champaign, United States of America

## Abstract

*Natronomonas pharaonis* is an archaeon adapted to two extreme conditions: high salt concentration and alkaline pH. It has become one of the model organisms for the study of extremophilic life. Here, we present a genome-scale, manually curated metabolic reconstruction for the microorganism. The reconstruction itself represents a knowledge base of the haloalkaliphile's metabolism and, as such, would greatly assist further investigations on archaeal pathways. In addition, we experimentally determined several parameters relevant to growth, including a characterization of the biomass composition and a quantification of carbon and oxygen consumption. Using the metabolic reconstruction and the experimental data, we formulated a constraints-based model which we used to analyze the behavior of the archaeon when grown on a single carbon source. Results of the analysis include the finding that *Natronomonas pharaonis*, when grown aerobically on acetate, uses a carbon to oxygen consumption ratio that is theoretically near-optimal with respect to growth and energy production. This supports the hypothesis that, under simple conditions, the microorganism optimizes its metabolism with respect to the two objectives. We also found that the archaeon has a very low carbon efficiency of only about 35%. This inefficiency is probably due to a very low P/O ratio as well as to the other difficulties posed by its extreme environment.

## Author Summary

Extremophiles are organisms that thrive in physically or geochemically extreme conditions that are detrimental, even lethal, to the majority of life on Earth. *Natronomonas pharaonis* is one that has been able to adapt to both high salt concentration and an alkaline pH. In this study, we investigate the chemical reactions that occur within the microorganism, collectively referred to as its metabolic network, that allow it to convert the nutrients in its environment to biomass and energy. Specifically, we reconstructed the network by collecting evidence for the existence of reactions from the literature, and then supplemented them with computational approaches, for example by searching the genome of *Natronomonas pharaonis* for genes that could potentially encode analogs of known enzymes from other organisms. Finally, with the network in hand, we developed a computational model which we used to simulate growth. Among other results, we found indications that *Natronomonas pharaonis* regulates its metabolism such that energy production and growth are maximized. Despite this however, we also found that *Natronomonas pharaonis* is only able to incorporate a very small fraction of the total carbon that it consumes (approximately 35%), likely in no small part due to the difficulties posed by its environment.

## Introduction

*Natronomonas pharaonis* is a polyextremophilic archaeon that can be isolated from soda lakes, where it has to cope with two extreme conditions: high salt concentration and an alkaline pH. The microorganism thrives at an optimal pH of 8.5, and remains viable up to a pH of about 11. Two strains of *Natronomonas pharaonis* have been described so far: strain Gabara from lake Gabara in Egypt (DSM 2160) [1], which was used in this study, and strain SP1 from lake Magadi in Kenya (DSM 3395) [2]. Among other results, we show that the microorganism is able to grow on a single carbon source, such as acetate, glutamate and pyruvate, unlike the more well-studied halophilic archaeon *Halobacterium salinarum*. This greatly simplifies several experimental protocols, in particular those based on stable isotope labeling. The combination of this capability, a completely sequenced genome [3] and the recent successful development of a transformation protocol (separate publication) will increase the prominence of *Natronomonas pharaonis* in the study of extremophilic life.

The metabolic network of an organism can be reconstructed at the genome scale through the combination of genomic, biochemical and physiological data, using bioinformatics methods and literature review [4]–[8]. The resulting network, comprised of the known and hypothesized reactions that take place within the organism, is valuable in that it forms a knowledge base of cellular metabolic capabilities. For example, inspection of the network would allow one to draw hypotheses regarding nutritional requirements and biosynthetic capabilities. Moreover, metabolic reconstructions can also serve as convenient starting points for crafting genome-scale, constraints-based models of metabolism, which allow more detailed computational/formal analysis. Indeed, constraints-based models have emerged as important alternatives to kinetic models because they do not require the detailed kinetic information needed by the latter. Rather, constraints-based models require only generally available physicochemical information such as stoichiometry, reversibility and energy balance [9]–[11], data which are already typically included in, or at least could easily be derived from, metabolic reconstructions. More sophisticated data, such as flux (reaction velocity) limits, could also be easily integrated. The repertoire of computational methods available under, or related to, the constraints-based framework include extreme pathways [12], elementary modes [13], and flux-balance analysis with its derivatives [14]–[16].

Genome-scale models of metabolism have been constructed and analyzed for various organisms [6], yielding interesting results, such as the prediction of *E. coli* metabolic mutant phenotypes up to an accuracy of 86% [17], a simulation and characterization of *E. coli*'s secreted metabolites under various nutrient and oxygenation conditions [14], a study of the methanogenic growth of *M. barkeri* [18], and an analysis of growth on a relatively complex medium for *L. plantarum* [19] and *Halobacterium salinarum* [20], [21]. In this work, we present a manually curated, genome-scale metabolic reconstruction for *Natronomonas pharaonis*, and report on the development and use of a constraints-based model from it. This is the third curated reconstruction for a microorganism belonging to the archaeal domain of life; the first being for *M. barkeri* [18] and the second for *Halobacterium salinarum* [20], [21]. We also report on the experimental determination of several parameters relevant to growth, including material uptake, respiratory rates, and the distribution of carbon in the biomass (e.g., amino acids, nucleotides, etc.).

## Results/Discussion

### The metabolic network

**Figure 1:**
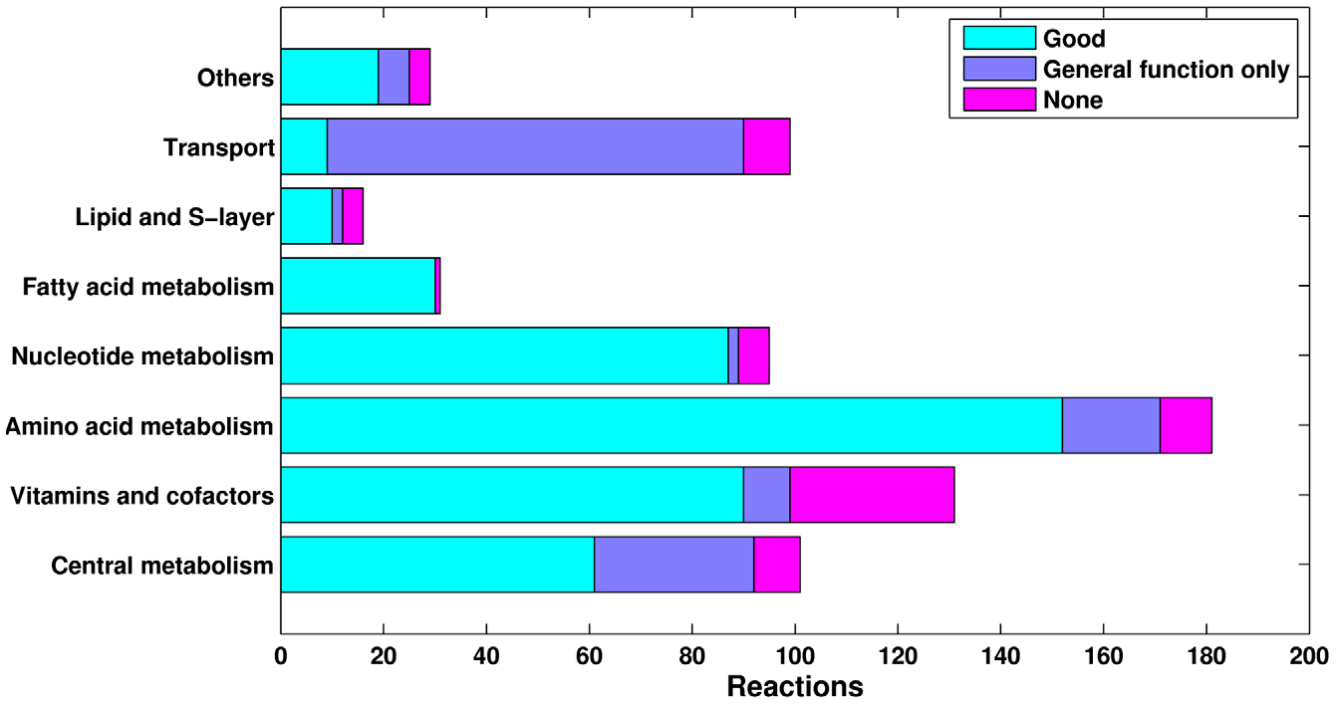
Metabolic network statistics. The reconstructed metabolic network for *Natronomonas pharaonis* is composed of 683 reactions, covering 654 genes, and 597 metabolites. The graph shows how the reactions are distributed among general functional categories, and also shows the numbers for which: (1) enzyme-coding genes could be reliably assigned; (2) only genes with general functional annotation (i.e., with unclear substrate specificity) could be associated; and (3) no genetic evidence could be found. Reactions with no associated evidence were added to the network in order to fill pathway “gaps”. Note that most transport reactions are labeled “General function only”. This is because it is generally very hard to assign substrate specificities to transporters using sequence analysis only.

The reconstructed *Natronomonas pharaonis* metabolic network is composed of 683 reactions and 597 distinct metabolites. It covers 654 genes, not including those with known transport function but with unclear substrate specificity. Reactions were added to the network based on either genetic (e.g., homologs of known enzyme-coding genes) or literature (e.g., enzyme assays, labeling studies) evidence. Figure 1 shows the distribution of the reactions based on the former type of supporting data. Specifically, it shows the number of reactions, grouped according to general functional categories, for which: (1) enzyme-coding genes could be reliably assigned; (2) only genes with general functional annotation (i.e., with unclear substrate specificity) could be associated; and (3) no genetic evidence could be found. Given the relatively recent isolation of *Natronomonas pharaonis*, its literature base, particularly for subjects relevant to metabolism, is still quite small. This is the reason why most reactions in the network only have bioinformatic support. Nevertheless, at least 168 (24%) of the reactions are associated with literature (experimental) evidence from related haloarchaeal species (e.g., *Halobacterium salinarum*). Note that reactions with neither genetic nor literature support were nevertheless added to the network in order to fill pathway “gaps”.

We obtained an initial draft of the metabolic network by merging the reaction database (LIGAND) from KEGG [22] with the *Natronomonas pharaonis* genome annotation found in Halolex [23], [24]. The latter resource is a genome information system that specializes in halophilic microorganisms. Because of the procedure used, most of the reactions in our reconstructed network are defined according to the definitions found in the LIGAND database. Nevertheless, the network also contains reactions that had to be manually defined, such as newly characterized pathways that are not yet contained in KEGG. This is particularly relevant since the archaea regularly use pathways that are different from, or modifications of, those studied in most model organisms, which are often from the bacterial domain of life, and it typically takes a while before these are reflected in databases. For example, it was recently shown that aromatic amino acid biosynthesis in *Methanocaldococcus jannaschii* does not use the classical precursors erythrose 4-phosphate and phosphoenolpyruvate, but rather proceeds via an alternative pathway that begins with the condensation of 6-deoxy-5-ketofructose 1-phosphate with aspartate 4-semialdehyde [25]. Genetic evidence suggests that the same (or a similar) pathway is in operation in *Natronomonas pharaonis*. This alternative pathway was still not in KEGG when we did our reconstruction. Another example is the modified mevalonate biosynthesis pathway that was also demonstrated in *Methanocaldococcus jannaschii* [26]. The modification to the classical pathway, essentially a change in ordering between a decarboxylation and a phosphorylation step, is still not reflected in KEGG. Again, genetic evidence suggests that this pathway is also in operation in *Natronomonas pharaonis*. The reconstructed network is provided as supplementary information, both in tabular (Table S1) and SBML format (Text S2).

Although in silico gene deletion simulations were not part of our analysis, information on putative logical relationships between genes and reactions was nevertheless included in the reconstruction. For example, the oxidation of pyruvate to acetyl-CoA and CO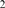 is catalyzed by the polymeric enzyme, pyruvate-ferredoxin oxidoreductase (EC 1.2.7.1). This protein consists of two distinct subunits, which in *Natronomonas pharaonis* are encoded by NP4044A (beta subunit) and NP4046A (alpha subunit). Accordingly, the reaction was assigned the gene-logic formula *NP4044A* 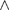 *NP4046A*, because, presumably, both subunits are required in order for the enzyme to function. We should note, however, that given the current lack of appropriate genomic data, such as genome-scale single deletion lethality assays, the logical relationships were mostly inferred only from the genome annotation (e.g., keywords such as “subunit” or “component” in the case of complex enzymes). Accordingly, this information should be taken with a grain of salt. For example, four genes are currently annotated in the genome as “probable aspartate aminotransferase (EC 2.6.1.1)”, and accordingly appear as disjuncted terms in the gene-logic of the reaction catalyzed by the enzyme EC 2.6.1.1:
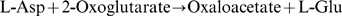



However, even if all four genes are indeed aspartate transaminases, there is a good probability that not all of them actually catalyze the reaction defined above. Some could work with an acceptor different from 2-Oxoglutarate, and as such are not true isoenzymes.

All of the reactions in the reconstructed network are mass and charge balanced, except for 10. The latter could not be balanced because some reactants are still unknown. Seven of the reactions are involved in cofactor biosynthesis, and the rest belong to amino acid degradation pathways. The ionization state of the metabolites reflected in the network is that of the most abundant microspecies under a pH of 9.0.

### Analysis of biomass composition

**Figure 2:**
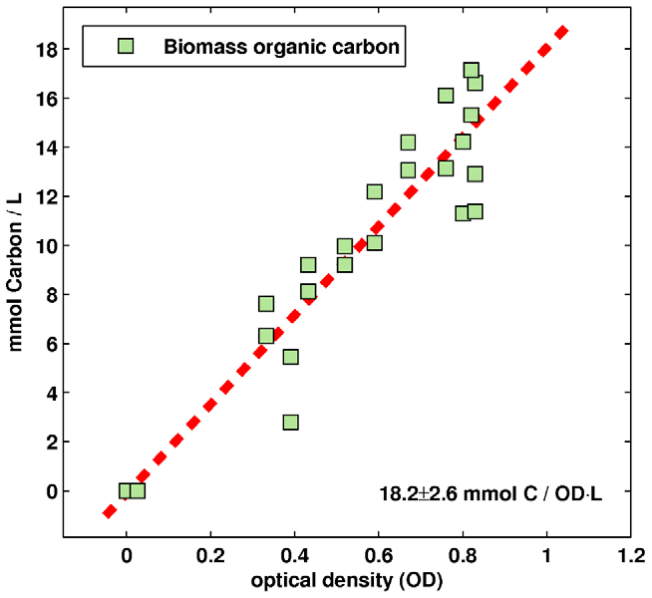
Biomass total organic carbon content. The total organic carbon content of the biomass was measured at different optical densities. We calculated a linear correlation of 18.2
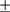
2.6 mmol of carbon per OD

In flux balance analysis, growth is typically simulated through the use of a growth reaction, which is a pseudo reaction where the reactants are the components of the biomass in the proper ratios and the product is a unit of population (e.g., mg DW, OD

ml). Clearly, a prerequisite for the definition of this reaction for *Natronomonas pharaonis* is an approximation of the microorganism's average biomass composition, at least under the conditions used. For this we determined the total organic carbon content of samples taken from cultures at different population levels (optical densities). The data is summarized in Figure 2. Overall, we calculated a linear correlation of 18.2
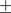
2.6 mmol organic carbon per OD

L, compared to 23.1 mmol per OD

L in the closely related halophile *Halobacterium salinarum* [21].

L. This value was used to formulate the growth function used for flux balance analysis (see text for details).

**Figure 3:**
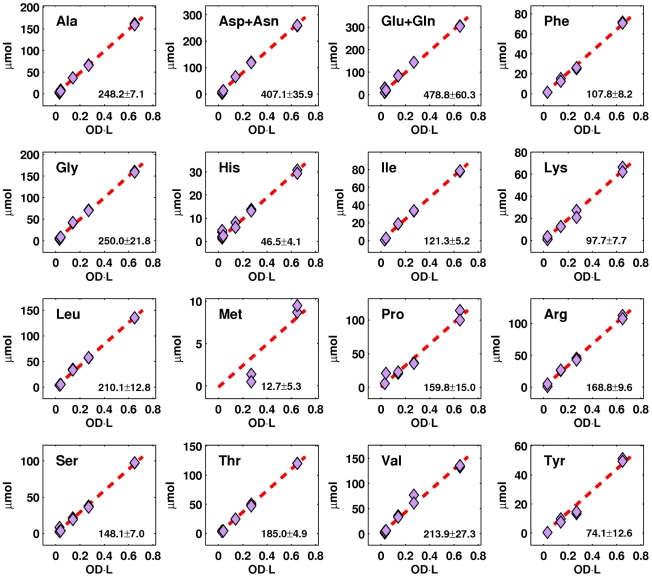
Amino acid composition of the biomass at different optical densities. The values represent total cellular content, including protein residues and free metabolites. The good linear correlations imply that the average (normalized) cellular content of each amino acid remains reasonably constant throughout growth. Due to experimental limitations, only combined values could be obtained for aspartate and asparagine as well as for glutamate and glutamine. Measurements for cysteine and tryptophan could not be reliably obtained.

Determination of the total organic carbon content of the biomass is only the first step. The next is to decompose it into the different building blocks. Given that proteins typically account for a large fraction, if not a majority, of the organic mass of most microorganisms, we began our characterization of the *Natronomonas pharaonis* biomass by analyzing its amino acid content. Specifically, we took detailed quantitative measurements of the amino acid composition of cell cultures at different population levels (Figure 3; see *Growth on Acetate*). From these measurements, we were able to observe good linear correlations between the amino acids and the optical density, which implies that the average cellular amino acid composition remains reasonably constant throughout growth. This observation reinforces the validity of using a static definition of growth in flux balance models, such as the one used in this study. Note that due to experimental limitations, measurements for aspartate and glutamate are already inclusive of asparagine and glutamine, respectively, and cysteine and tryptophan could not be reliably determined. For these amino acids, statistical analysis of the predicted *Natronomonas pharaonis* proteome was used to specify coefficients in the growth reaction (see *Growth on Acetate*).

The amount of amino acids in the biomass added up to about 335 mg/OD

L, which represents approximately 75% of the total organic mass or 80% of the total organic carbon content. The reason for this very high amino acid (protein) composition is unclear. A similar relationship between the amino acids and the organic mass was also observed in *Halobacterium salinarum* (data not shown).

2-Sulfotrehalose has been demonstrated to be an osmolyte used by several haloalkaliphilic archaea, including *Natronomonas pharaonis* [27]. This molecule was reported to accumulate in amounts over 2.5 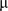
mol per mg protein [27], which translates to about 855
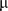
g of the compound per mg protein. Clearly, such a high intracellular concentration of 2-sulfotrehalose can be excluded under the current conditions, given that proteins already account for 75% of the total organic mass. Thus, the likely scenario is that under the conditions used, cells rely predominantly, if not completely, on inorganic ions to counter osmotic stress; a situation that has already been observed for *Natronomonas pharaonis* under other conditions [27]. Indeed, using NMR to analyze intracellular solutes (see *Detection of 2-Sulfotrehalose*) we were not able to detect any dissacharide.

In addition to amino acids, the cellular biomass is also made up of other molecules such as lipids (e.g., archaeaol), sugars (e.g., S-layer glyco moieties), cofactors (e.g., NAD/P, coenzyme-A, retinal), and other small molecules. Due to lack of experimental data, the stoichiometric coefficients that we used for these molecules in the growth reaction were simply taken from the approximations used in the *Halobacterium salinarum* model [21], calculated in proportion to the ratio of the measured amino acid contents of the two halophiles. Finally, the remaining amount of organic mass (from the total organic carbon analysis) was assumed to comprise of nucleotides (approximately 20%). Separate coefficients for the different nucleotide molecules were calculated in proportion to the 63.4% GC content of the genome. The final growth reaction we used in our model is shown in Table 1.

**Table 1:** Major components of the *N. pharaonis* biomass.

Molecule	Amount^a^	Molecule	Amount^a^
	( 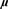 mol/OD  L)		( 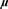 mol/OD  L)
Amino Acids		Nucleotides^c^	
Ala	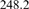 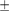 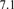	AMP+dAMP	81.6
Cys	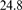	UMP+dTMP	81.6
Asp+Asn	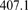 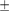 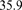	GMP+dGMP	47.1
Glu+Gln	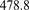 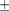 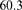	CMP+dCMP	47.1
Phe	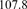 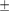 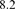		
Gly	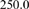 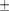 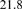	S-Layer non AA^d^	
His	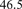 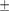 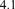	GalNAc	2.2
Ile	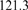 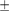 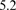	GlcNAc	2.2
Lys	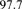 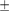 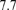	Gal	9.6
Leu	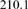 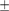 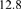	Glc	9.6
Met^b^	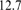 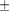 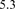		
Pro	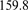 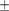 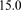	Membrane^d^	
Arg	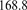 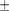 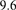	Archaeol	20.0
Ser	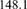 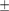 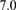		
Thr	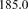 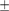 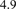	Others	
Val	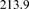 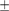 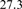	ATP	2.0
Trp	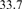	Na 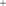 (K 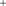 )	2813.4
Tyr	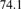 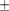 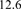	Cl 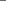	2813.4

^a^Error margins are provided for experimentally determined values.

^b^Very low value probably due to loss incurred during hydrolysis.

^c^Calculated as the total organic carbon minus the other components.

^d^Taken in proportion to *H. salinarum* values [20].

### Aerobic growth on a single carbon source

It was previously believed that any growth medium for *Natronomonas pharaonis* would require the presence of leucine because of a disruption in the 2-isopropylmalate synthase gene (NP2206A) [3], which is involved in the biosynthesis of the amino acid. However, in the course of optimizing the carbon sources supplied in the medium, which we undertook in the interest of designing stable isotope experiments, we observed that the haloalkaliphile was able to grow on a medium that contained just acetate. Subsequent resequencing of the 2-isopropylmalate synthase gene showed that the gene is still interrupted, so the reason for the phenotype is uncertain. Nevertheless, it is now clear that *Natronomonas pharaonis* is capable of growing (aerobically) on a single carbon source. Indeed, although the rest of this study deals with growth on acetate, we were able to find other possible substrates, including glutamate and pyruvate.

In order to simulate aerobic growth on acetate using the constraints-based model, three parameters needed to be further defined: (1) the consumption rate of acetate, (2) the consumption rate of oxygen, and (3) the maintenance energy. Under certain assumptions however (see *Computational Analysis* for details on the calculations), it turns out that what is critical to the first two parameters with respect to the analysis is just the ratio between the two, i.e., the ratio between acetate and oxgyen usage. Scaling the actual values simply results in a similar scaling of the output of the model (in this case, the growth flux). Accordingly, for most of the analysis we could simplify the calculations by reducing the parameter space to just two instead of three; namely, the acetate to oxygen consumption ratio and the maintenance energy.

The model, through the definition of the growth reaction, already accounts for the energy involved in synthesizing the basic building blocks of the biomass (see Table 1 for a complete list) from the supplied materials. This is accomplished in an implicit manner based on the pathways defined in the metabolic network. However, there are numerous other energy-consuming processes performed by cells, such as assembling the building blocks into larger structures, motility, repair, cellular division, etc. These other processes are represented by the maintenance energy parameter.

**Figure 4:**
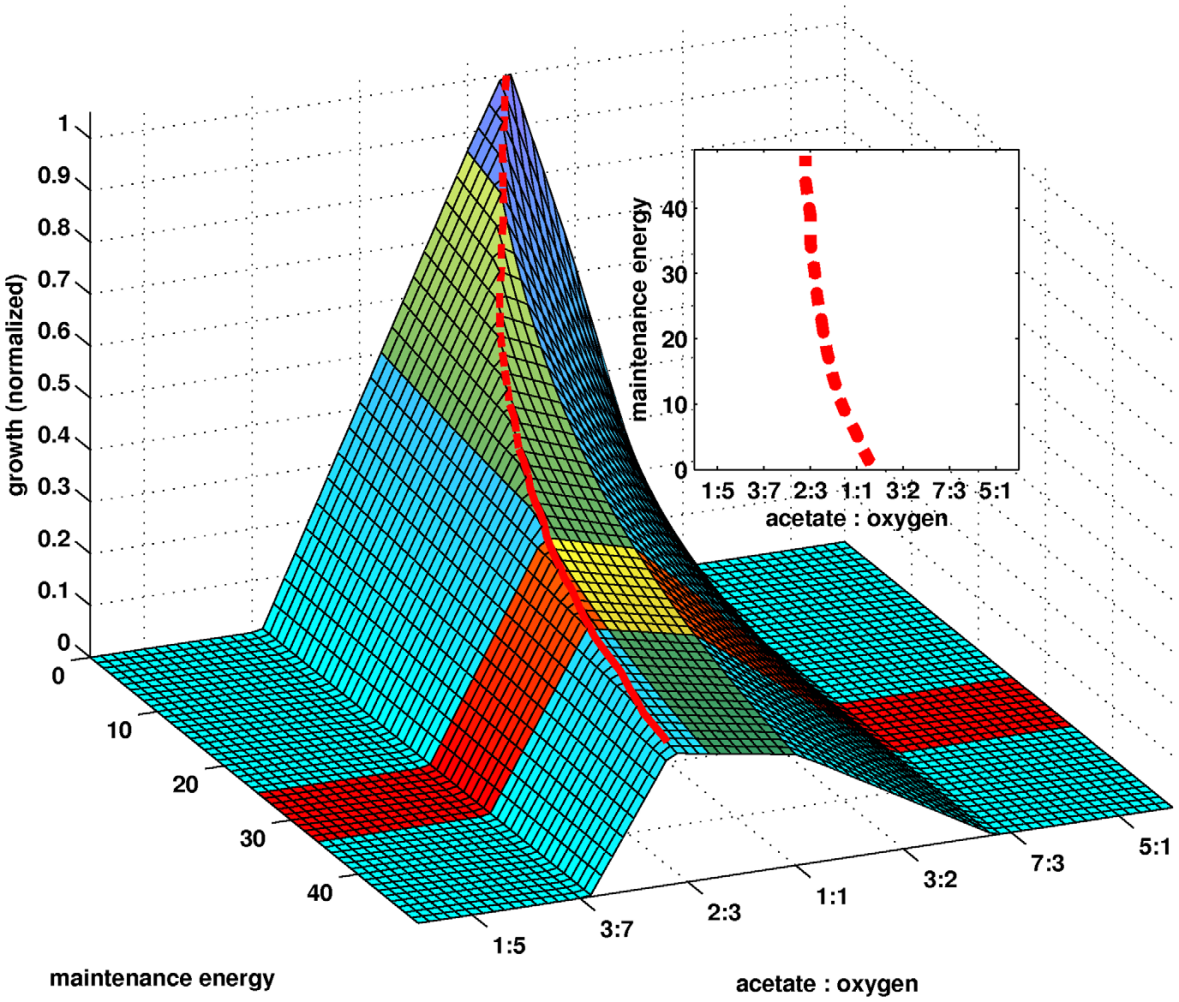
Theoretical analysis of aerobic growth on acetate. The surface represents the theoretical maximum growth of *Natronomonas pharaonis* as a function of two parameters: (1) the acetate to oxygen ratio and (2) the maintenance energy (in 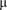
mol ATP per 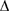
OD

The theoretical maximum level of growth as a function of the acetate∶oxygen parameter and the maintenance energy is plotted in Figure 4. The latter parameter is defined in terms of the equivalent 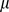
mols of ATP hydrolized in producing an OD

ml worth of biomass (i.e., 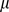
mols of ATP per 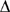
OD

ml). It is immediately recognizable from the figure that growth is not possible for a very high (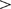
7∶3) or a very low (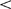
3∶7) acetate to oxygen consumption ratio. Indeed, the model clearly indicated that having such ratios is physiologically impossible. Formally speaking, the linear programs do not have feasible solutions under such settings of the parameter. In the case where the ratio is too high, i.e., too much acetate, energy cannot be produced in amounts sufficient to process all of the consumed material. Note that all of the carbon (acetate) taken up by cells will have to be either: (1) incorporated into the biomass; (2) secreted as the respiratory byproduct CO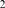; or (3) secreted as some other metabolite after conversion. Accordingly, if the acetate to oxygen consumption ratio is too high, enough energy that would allow any distribution of the consumed carbon into the possible fates simply cannot be generated. In an analogous way, a very low acetate to oxygen consumption ratio is also impossible because under such conditions, there is not enough organic material that can be oxidized in order to allow the conversion of all the consumed oxygen to H
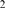
O.

ml). The green shaded region corresponds to experimentally observed values of the former (i.e., acetate∶oxygen), while the orange shaded region corresponds to experimentally determined values of the latter (i.e., maintenance energy). Clearly, for different values of the maintenance energy, theoretical maximum growth is achieved at different acetate to oxygen ratios. This optimality relationship is represented by the red broken curve. A projection of this into the x,y-plane is shown in the inset.

Optimal acetate to oxygen consumption ratios as a function of the maintenance energy are shown in Figure 4 using the red broken curve (see also figure inset). They are optimal in the sense that they are the consumption ratios that would theoretically allow the highest level of growth for the different values of the maintenance energy (i.e., the amount of energy required to produce a unit of biomass) parameter. Notice that as the maintenance energy parameter is increased, the optimal acetate to oxygen ratio decreases. This is because under the conditions used, energy can only be derived through the oxidation of acetate. Thus, increasing the amount of energy needed to produce a unit of biomass means that a greater fraction of the consumed carbon (acetate) will have to be converted into energy. In addition, oxidization of a greater amount of acetate would require a greater amount of oxygen, and this also decreases the optimal ratio.

It is clearly of interest to compare how the theoretical results summarized in Figure 4 compare to the real-world behavior of *Natronomonas pharaonis*. Accordingly, we prepared actual cultures where we grew the archaeon aerobically on acetate. The experimental data is summarized in Figure 2 of Text S1 (supplementary information). During the early stages of growth, we observed an acetate∶oxygen comsumption ratio of about 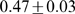. This is represented in Figure 4 by the green shaded region. In addition, we also approximated the maintenance energy by calculating the amount of oxygen consumed per unit increase in population size (optical density), and then multiplying the result with a P∶O ratio of 1∶1. This calculation resulted in a maintenance energy of about 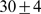 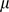
mols ATP per 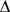
OD

ml, which is represented in the figure by the orange shaded region.

Not much is currently known regarding the biochemistry of the respiratory chain of *Natronomonas pharaonis*. Nevertheless, the pathway has been demonstrated to be functional, by showing increases in both ATP level and membrane potential in response to aeration [3]. With respect to the genome, complete sets of ORFs encoding analogs of Complex II and Complex IV genes are present, but none could be assigned for Complex III. Homologs of most Complex I subunits are present, but subunits comprising the NADH acceptor module (nuoEFG) could not be assigned. Indeed, NADH dehydrogenation likely occurs via a non-proton pumping type II NADH dehydrogenase NP3508A, which is a homolog of the *Acidianus ambivalens ndh* gene [28]. However, note that the presence of the type II NADH dehydrogenase does not preclude the possibility of another donor molecule for which proton translocation is possible. Because of the relatively scarce information available for *Natronomonas pharaonis*, the P∶O ratio that we used to calculate the maintenance energy above is actually the experimental value for the closely related *Halobacterium salinarum*, which was determined using oxygen pulse experiments [29], [30]. Thus, the maintenance energy value reported above is still subject to scaling. Nevertheless, interpreting it in terms of the O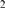 consumed per unit increase in biomass (i.e., 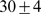 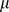
mols O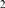 per 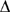
OD

ml), instead of ATP, should represent a fairly accurate approximation of the parameter.

The intersection of the regions corresponding to the experimentally determined values of the acetate to oxygen consumption ratio (green region in Figure 4) and the maintenance energy (orange region) could be thought of as the area representing experimentally observed growth behavior (shaded yellow in the figure). It is immediately recognizable that this region is within the space that the model considers to be physiologically permissible with respect to the two parameters. However, what is more interesting is the fact that all of the points in this region are at least reasonably close to the optimality curve (red curve in the figure). This means that the cells must be using an acetate to oxygen consumption ratio that is at least near-optimal with respect to growth and energy production (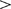
90% based on theoretical calculations), whatever the actual maintenance energy may be. Indeed, the region is located on a relatively flat area of the surface, where “near optimality” is robust with respect to the two parameters. While the observations above, by themselves, say nothing directly about the optimality of the actual fluxes used by the cells, it is at least an argument in favor of the hypothesis that *Natronomonas pharaonis*, under the conditions, optimizes its metabolism with respect to growth and energy production. Indeed, it has been demonstrated in *Escherichia coli* that under similar, simple growth conditions, a theoretical optimization of the fluxes with respect to energy provides a reasonable approximation of the actual fluxome [31], [32].

**Figure 5:**
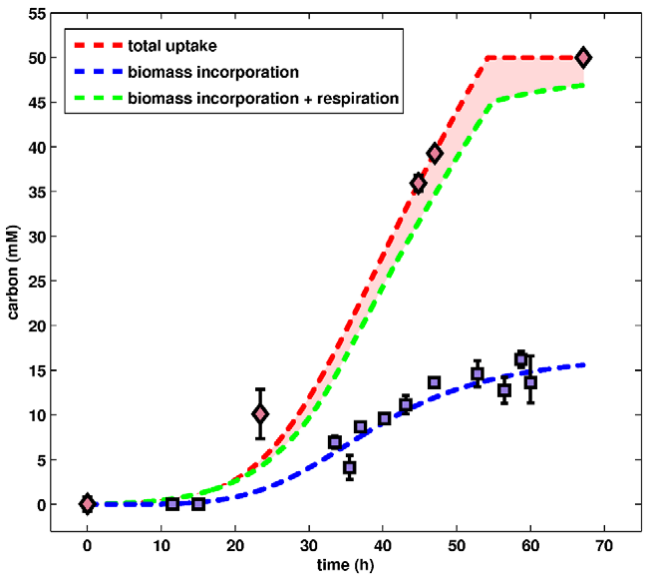
Analysis of carbon fate. *Natronomonas pharaonis* was grown on the single carbon source, acetate. Accordingly, the total amount of carbon that has been consumed (red curve) is simply twice the amount of acetate that has disappeared from the medium. Two possible fates for these consumed materials are incorporation into the biomass (blue curve) and excretion as the respiratory end product CO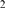. The total of these two possible fates, i.e., incorportion+respiration, is represented in the figure by the green curve. Accordingly, the delta region (red shaded) between total carbon consumption (red curve) and the sum of both fates (green curve) represents carbon that was consumed but not accounted for by incorportion or respiration. While this delta region could potentially be due to carbon being secreted in some other form, it is more likely that this difference is due to small methodological inaccuracies (see text for more details), and that incorporation and respiration fully account for carbon consumption. Under the conditions used, *Natronomonas pharaonis* showed a very low carbon incorporation rate of approximately 35%.

We mentioned earlier that all of the carbon consumed by cells will have one of the following three fates: (1) incorporated into the biomass; (2) secreted as the respiratory byproduct CO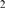; or (3) secreted as some other metabolite after conversion. Given that only acetate was supplied in the medium, then the only other possible way through which the cells could incorporate carbon is through CO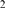 fixation. This capability is suggested by the presence of various enzymes in the genome of *Natronomonas pharaonis*, so we checked if it is indeed a contributing factor under the conditions used. Specifically, we supplied 13C sodium carbonate in the medium, and then tried to see if label would turn up in amino acids (which represent 75% of the biomass). Results of the stable isotope experiments showed that no net fixation seems to occur (data to appear in a separate publication). Accordingly, the amount of acetate that disappears from the medium is a direct measure of the total carbon consumption of the cells (i.e., two carbon atoms per acetate molecule). By correlating this consumption, which is represented as “total uptake” (red borken curve) in Figure 5, with the total amount of carbon in the biomass (see *Analysis of biomass composition*), we find that only about 35% of the total carbon consumed was incorporated into the cells (i.e., fate 1; blue curve in Figure 5). This very low carbon efficiency is likely due to a very small P/O ratio as well as to the other difficulties presented by the extreme environments of the microorganism.

The respiratory exchange ratio (RER) is the ratio between the production of CO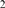 and the consumption of O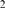. This ratio can be theoretically calculated for the complete oxidation of any metabolite. In the case of acetate (and some sugars in general), the RER is typically close to one. We used this fact to approximate the CO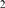 production of our cultures, specifically by multiplying the experimentally determined oxygen consumption with it. We found respiratory-related CO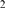 production to account for about 63% of the total carbon consumption.

The sum of fates 1 (incorporation) and 2 (CO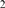 production) is plotted (green curve) in Figure 5 to provide a comparison with the total carbon consumption (red curve). Clearly, the difference between this sum and the total carbon consumption corresponds to material that was consumed but neither incorported into the biomass nor oxidized to CO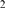. While this relatively small difference, which is represented in the figure by the red shaded region, could potentially be due to carbon being secreted in some other form (i.e., fate 3), it is more likely to simply be due to small methodological inaccuracies, such as deviations in the actual RER ratio or errors in the measurements. That is, under the conditions used, biomass incorporation and CO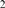 production together likely fully account for carbon consumption.

### Conclusions and outlook

We presented a genome-scale, manually curated metabolic reconstruction for the polyextremophile *Natronomonas pharaonis*. The reconstruction itself represents a summary of the knowledge regarding the haloalkaliphile's metabolism. As such, it would greatly assist future investigations on archaeal pathways. This is particularly relevant since the archaea are known to, quite frequently, use novel or modified pathways from those in existing model organisms [24]. An existing knowledge base that gives an overview of metabolism, such as this reconstruction, could, for example, assist in identifying knowledge “holes” or “gaps”, which are promising directions for further study. Indeed, this is very timely as research on haloarchaeal metabolism, up until recently, has been limited by the experimental protocols available for its model organisms. For example, while genetic transformation was possible for *Halobacterium salinarum*, the microorganism required multiple carbon sources in the medium, and this complicated other protocols such as stable isotope labeling. In the case of *Natronomonas pharaonis*, work was limited by the absence of a procedure for genetic modification. Accordingly, the recent development of a transformation protocol for *Natronomonas pharaonis* (separate publication) means that we now have a haloarchaeon for which genetic manipulation is possible and labeling experiments are relatively simple.

In order to complete a constraints-based model for *Natronomonas pharaonis*, we experimentally determined several physiological parameters. These include a characterization of the biomass composition, and a quantification of carbon and oxygen consumption under typical conditions. The data was integrated with the metabolic reconstruction to create a computational model that we used to analyze the behavior of *Natronomonas pharaonis* when grown on a single carbon source. Among other results, we found that the archeaon, when grown aerobically on acetate, uses an acetate to oxygen consumption ratio that is theoretically near-optimal with respect to growth and energy production. This supports the hypothesis that, under simple conditions, *Natronomonas pharaonis* optimizes its metabolism with respect to these two objectives. We also found that the archaeon has a very low carbon efficiency of only about 35%, likely due to a very low P/O ratio and the other difficulties brought about by the haloalkaliphilic character of its environment.

Stable isotope labeling of small-molecules (e.g., 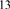
C-acetate in this case) is a powerful tool for characterizing metabolic pathways. However, this approach requires detailed information on the fates of individual atoms in each reaction, and these can be very difficult to collect in a useful form. For this reason, one of the things that we are currently working on is to add carbon fate data to the metabolic network reconstruction. Our eventual goal is to elucidate some of the currently unclear archaeal pathways, for example to identify the exact precursors of archaeal aromatic amino acid biosynthesis [25], and to directly measure the internal fluxes of *Natronomonas pharaonis* [31], [32], such as in response to different stimuli.

## Materials and Methods

### Metabolic reconstruction

A prior partial reconstruction of the *Natronomonas pharaonis* metabolic network was downloaded from the PATHNET database of the Halolex system [23], [24]. We used this as the starting point of our reconstruction. Evidence used to support the inclusion of a reaction/enzyme into the network is of two types: bioinformatic evidence, e.g., high similarity to a known enzyme; and literature evidence, such as experimental reports of enzyme assays, labeling studies, and nutrient uptake (for transporters). Pathway gaps, i.e., reactions devoid of literature or genetic indicators but belonging to pathways that seem to be present in the microorganism, were added to the network regardless (and marked accordingly), particularly if they are involved in the production of a compound essential to growth. Since the current body of metabolic literature for *Natronomonas pharaonis* is still quite small, data from closely related microorganisms was used, particularly from the intensively studied halophile, *Halobacterium salinarum*.

Reaction reversibilities were taken from literature sources whenever available. Particularly helpful in this regard is the BRENDA database [33], [34], which, among other things, stores reversibility information from various organisms. In the absence of such data, we used the assignments made in existing reconstructions as well as those made by Ma and Zeng [35], who annotated the reversibility of reactions defined in KEGG [22] based on biochemical principles. As a consequence of the reconstruction procedure that we used, most of the reactions in the network are based on the definitions in KEGG. However, a number had to be manually added because some reactions, particularly those from newly characterized archaeal pathways, still do not exist in the database. Moreover, KEGG reactions had to be modified in order to correct errors or to achieve elemental and charge balance. ORFs with a known general function but unclear substrate specificity were linked to all the possible reactions that they could affect (marked accordingly). Microspecies distributions with respect to ionization states at pH 9.0 were approximated using the pKa Plugin for Marvin (Marvin 5.3.0, 2009, ChemAxon; http://www.chemaxon.com). The reader interested in more information on metabolic reconstructions is directed to the reviews by [5] and [4].

### Computational analysis

A metabolic network can be conveniently represented by a stoichiometric matrix 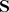, where each column corresponds to a reaction and each row to a metabolite. The entries of 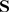 are the stoichiometric coefficients that define the relationships between the reactions and compounds. A positive value for 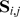 indicates that compound 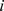 is produced in the left to right direction of reaction 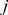, while a negative value indicates that it is consumed. A set of fluxes that is both consistent with the known constraints and optimal with respect to some objective function can be obtained by solving the linear program
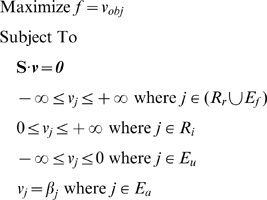
(1)where 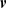 is a vector of fluxes defining the flux 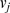 through each reaction 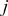, 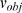 is the objective function, 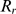 is the set of reversible internal reactions, 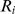 is the set of irreversible internal reactions, 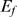 is the set of exchange fluxes associated with ubiquitous metabolites, and 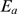 is the set of exchange fluxes that correspond to metabolites that were treated as parameters during analysis (e.g., acetate and oxygen uptake). The set of ubiquitous compounds that we used include CO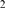, H
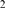
O, Na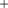, Cl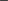, K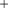 and H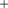. For the purpose of the analysis, sulfate, orthophosphate, as well as a number of other ions were also assumed to be available in abundance. To account for the possibility that cells produce and accumulate certain metabolites in the medium, for example in the case of overflow metabolism, the set of one-way exchange reactions, 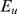, was included.

In the analysis of growth with respect to the acetate∶oxygen consumption ratio and the maintenance energy, Equation 1 was solved for different combinations of the two parameters using the growth reaction as objective function (see *Analysis of biomass composition*). Specifically, the acetate∶oxygen consumption ratio was allowed to vary from 1∶9 to 9∶1, and the maintenance energy was allowed to vary from 0 to 100 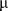
mol ATP per 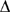
OD

ml. Most of our calculations were carried out using Matlab (TheMathworks, Massachusetts, USA). However, the default linear programming package was unable to handle our network due to its size, so we had to integrate the GNU Linear Programming Kit (GLPK) to handle flux analysis.

### Growth on acetate

For the analysis of growth, *Natronomonas pharaonis* strain Gabara (DSM 2160) was grown under aerobic conditions using chemically-defined media prepared according to Table 1 of Text S1 (supplementary information). Preparatory cultures were grown in 100 ml flasks containing 35 ml of the medium, from which inoculants were taken to start successive cultures. This was done repeatedly to adapt cells to the growth conditions. All cultures were prepared in flasks which had side arms to measure turbidity (cell density) via a Klett photometer, and were carried out at least in duplicates. Cell suspensions were shaken at 105 rpm at 40

C in the dark. At specific points during the growth period, samples were collected, and these were stored at −20

C. To separate the cells from the medium, samples were centrifuged for five minutes at 15,000 rpm, using a SS34 rotor. Amino acid analysis was performed on the pellets to determine the amino acid composition of the biomass, using a Biotronik LC 3000 analyser (Biotronik, Maintal, Germany). Oxygen saturation in the medium was monitored using the “Fibox 3-trace v3, fiber-optic oxygen meter” from Precision Sensing GmbH (Regensburg, Germany). Details on the calculation of actual oxygen consumption are provided as supplementary information (Text S1).

Due to experimental limitations, measurements of the aspartate and glutamate content of the biomass were already inclusive of asparagine and glutamine, respectively. For each of these pairs, specific coefficients in the growth reaction were assigned by dividing the combined total according to the relative abundance of the correspoding amino acids in all protein-coding genes (calculated assuming one copy per gene). Similarly, the individual contributions of cysteine and tryptophan to the biomass were approximated using their relative abundance compared to the rest of the amino acids (for which measurement was possible).

The formulation of the growth medium (Table S4) used in the analysis of aerobic growth is the result of optimizing previous synthetic media used for *Natronomonas pharaonis*. In particular, we tested different carbon sources, including all of the amino acids, acetate, glycerol, citrate cycle intermediates, and combinations thereof, in different concentrations using 96-well microtiterplates (Greiner bio-one).

The total organic carbon (TOC) content of the *Natronomonas pharaonis* biomass was determined by adapting a procedure typically used to determe the TOC of soils. The following solutions were used: *Solution 1* was prepared by dissolving 12.5 grams of silver sulfate in 40 ml of H
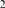
SO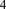; *Solution 2* was prepared by dissolving 98.08 grams of K
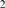
Cr
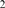
O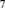 in deionized water to a total volume of 1 L; and *Solution 3* was prepared by dissolving 11.2 grams of FeSO
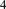
*7H
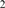
O in deionized water containing 6 ml of concentrated H
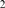
SO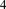 to a final volume of 400 ml. The procedure is as follows: the precipitate of each sample was collected after centrifugation, and this was resuspended in 500 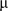
L of deionized water. Next, 1.5 ml of *Solution 1* was added in order to precipitate Cl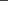 with silver. This step is necessary because Cl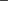, which is abundant in the biomass, interferes with a reaction downstream of the process. The resulting solution was centrifuged for 30 minutes, and 1 ml of the supernatant was transferred to a reaction vial for the succeeding steps. After adding 500 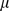
L of concentrated H
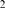
SO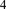 and 500 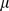
L of *Solution 2* to the vial, the solution was heated to 135

C, and then set aside to cool slowly. Once cooled to room temperature, the remaining amount of K
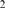
Cr
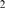
O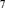 (added with *Solution 2*), which oxidizes the organic carbon in the biomass, was then determined via titration with *Solution 3*. This value is inversely related to the amount of carbon in the biomass. Titration was carried out using the *TitroLine easy* automatic titrator from Schott Instruments (Mainz, Germany).

### Detection of 2-Sulfotrehalose

*Natronomonas pharaonis* was grown in larger volumes (200 ml) to mid-log phase (OD 0.3–0.4), after which cells were harvested by centrifugation (6000 g, 30 min). Cell pellets were washed three times with 3.4 M NaCl, and then dried in a speed vac centrifuge. The pellets were suspended in 1.5 ml 70% (v/v) EtOH and vortexed for 5 min. Sonication for 5 min in a water bath followed, before centrifugation at 11 000 g for 10 min at 4

C in an Eppendorf microcentrifuge. The extraction step was repeated twice, and the ethanolic supernatants were pooled in a small pointy glas flask. Most of the solvent was removed by rotary evaporation, and final sample drying was achieved by centrifugation in a speed vac. For NMR analysis, samples were redissolved in 160 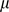
l D
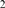
O (99.9%) and centrifuged for 10 min at 10 000 g in an Eppendorf centrifuge. A thin top layer was discarded, and the rest of the aqueous supernatant taken for measurements.

A 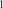
H WALTZ-decoupled 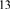
C-NMR spectrum was recorded using a Bruker DRX 500 spectrometer at 500 MHz with a 5 mm TXI probe head. Acquisition parameters included 33.3 kHz sweep width, 32 k datapoints, 90 pulse angle, 100 k transients and a temperature of 298 K. Chemical shifts were measured relative to dioxane (67 ppm).

## Supporting Information

Table S1 Reconstructed network in tabular format.
(0.39 MB XLS)

Text S1 Supplementary information.
(0.09 MB PDF)

Text S2 Reconstructed network in SBML format.
(0.51 MB XML)
