# Supplementary material for: Characterization of Growth and Metabolism of the Haloalkaliphile Natronomonas pharaonis
Source: PLoS Comput Biol. 2010 Jun 3;6(6):e1000799. doi: 10.1371/journal.pcbi.1000799 (PMC2881530; doi:10.1371/journal.pcbi.1000799)
Supplement: Text S1 — Supplementary information. (0.09 MB PDF) [file pcbi.1000799.s002.pdf]

Text S1 - Supplementary Information

For: Characterization of growth and metabolism of the haloalkaliphile *Natronomonas pharaonis*

By: Gonzalez *et al.*

Determination of oxygen consumption

The amount of dissolved oxygen in the medium (solution) was continuously monitored using the "Fibox 3-trace v3, fiber-optic oxygen meter" from Precision Sensing GmbH (Regensburg, Germany). Under conditions identical to those used in the aerobic cultures, argon was blown into a flask that contains only medium, until oxygen saturation dropped to zero. At that point, we removed the argon source and equilibrated the flask with air. We then used the rate at which oxygen dissolved into the medium to characterize oxygen dissolution kinetics. Specifically, the oxygen transfer rate (OTR) was defined as

$$OTR = \frac{dx}{dt} = k\,(x_{max} - x) \tag{1}$$

where x is the amount of dissolved oxygen, and x_{max} is the maximum value of x, which indicates saturation. The data and the fitted model are shown in Figure 1. With the appropriate parameters for Equation (1), we then calculated the oxygen consumption rate of a culture at time t using

$$consumption(t) = k\,(x_{max} - x) + m \tag{2}$$

where m is the current rate at which x is changing in the culture. For the sake of completeness, we note that Equation (1) has the analytical solution

$$x = x_{max} - (x_{max} - x_0)\,e^{-kt} \tag{3}$$

where $x(0) = x_0$ is the initial condition. In our calculations, oxygen saturation was taken to be 2.18 mg/L or 13.6 μmol/L (taken from *Int. J. Salt Lake Res.* 1:1-6).

Table 1: **Composition of defined medium used for *Natronomonas pharaonis*[a]**

Description	Value	Description	Value
NaCl	3.4 M	$FeSO_4$ x 7 H_2O	5 μM
Na_2CO_3	175 mM	$CuSO_4$ x 5 H_2O	4 μM
KCl	27 mM	$MnCl_2$	4 μM
Na_2HPO_4 x H_20	2mM	$CaCl_2$ x 2 H_2O	3 μM
NaH_2PO_4 x 7 H_2O	2mM	$ZnSO_4$ x 7 H_2O	3μM
$MgSO_4$ x 7 H_2O	1mM	Na-Acetate	25 mM
NH_4Cl	12mM		

[a]pH was adjusted to 9.2

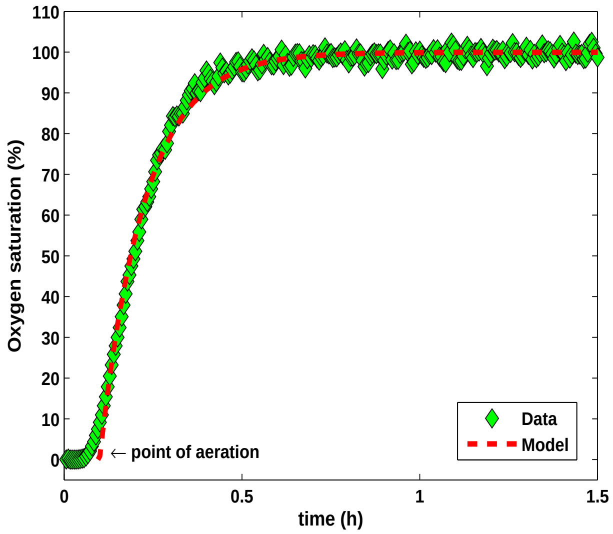

Figure 1: Dissolution kinetics of oxygen. The graph shows the velocity at which oxygen dissolves into the growth medium (cell-free) under the conditions used. This information was used to calculate the respiratory rate of cells during growth.

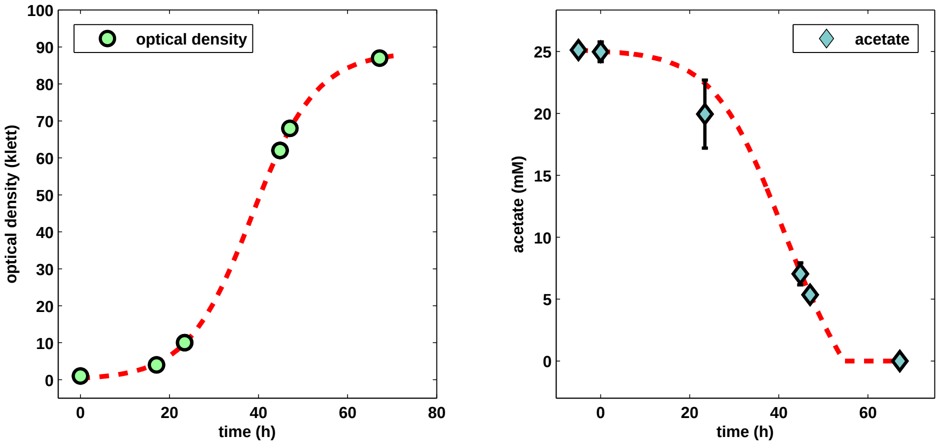

Figure 2: Growth on acetate. *Natronomonas pharaonis* was grown aerobically on a single carbon source: acetate. The graph on the left shows the population level (OD) and the graph on the right shows the depletion of acetate in the medium. Acetate concentrations were measured at different time points, at least in duplicate (error bars provided), using the K-ACETRM 10/06 assay kit from Megazyme (Wicklow, Ireland). The red broken curves represent fitted models.
